# Adequacy of Prenatal Care and Ensuing Maternal and Neonatal Severe Morbidity and Mortality

**DOI:** 10.1111/1471-0528.70119

**Published:** 2025-12-17

**Authors:** Zeenat Ladak, Jennifer A. Jairam, Sarah Swayze, Jennifer Shuldiner, Olesya Falenchuk, Richard Volpe, Noah M. Ivers, Joel G. Ray

**Affiliations:** ^1^ University of Toronto Toronto Ontario Canada; ^2^ Women's College Hospital Institute for Health System Solutions and Virtual Care Toronto Ontario Canada; ^3^ St. Michael's Hospital Toronto Ontario Canada; ^4^ ICES Toronto Ontario Canada; ^5^ Women's College Hospital Toronto Ontario Canada

**Keywords:** infant, maternal, morbidity, mortality, newborn, pregnancy, prenatal care

A mother or her infant may face life‐threatening events during childbirth or shortly thereafter, which may be preventable [1]. Adequacy of prenatal care is one determinant of maternal and newborn outcomes [2]. This study assessed whether adequacy of prenatal care is associated with maternal and newborn morbidity and mortality after birth.

This population‐based, retrospective cohort study was completed using deidentified healthcare datasets, linked using unique encoded identifiers at ICES (Table S1). Included were hospital‐based singleton livebirths and stillbirths from 20 to 42 weeks' gestation, in Ontario, Canada, from April 1, 2012, to March 31, 2020 (Table S2).

Study variables were chosen a priori (Figure S1). The exposure was adequacy of prenatal care, determined using the Revised‐Graduate Prenatal Care Utilisation Index (R‐GINDEX), which includes the number of prenatal visits, newborn gestational age, and initiation of first‐trimester care [2]. It reflects adequacy on a five‐level scale: no care, inadequate, intermediate, adequate (reference), and intensive care. The primary maternal outcome was a validated composite of any severe *maternal* morbidity and mortality (SMM‐M) from the mother's delivery hospitalisation date and up to 42 days thereafter [3]. The primary infant outcome was a validated composite of any severe *neonatal* morbidity and mortality (SNM‐M) from the infant's birth and up to 27 days thereafter (Table 1) [4].

**TABLE 1:** Risk of severe maternal morbidity and mortality (SMM‐M) within 42 days after the index delivery hospitalisation discharge date (upper), and severe neonatal morbidity and mortality (SNM‐M) within 27 days after the index birth hospitalisation discharge date (lower), each by adequacy of prenatal care based on the R‐GINDEX.

			Relative risk (95% CI)	
Outcome	Exposure group	No. (%) with outcome	Unadjusted	Adjusted^a^, ^b^
Severe maternal morbidity and mortality (SMM‐M)	No prenatal care (*N* = 1923)	96 (5.0)	1.51 (1.20 to 1.90)	1.42 (1.13 to 1.77)
	Inadequate prenatal care (*N* = 29 118)	930 (3.2)	1.08 (1.01 to 1.16)	1.08 (1.01 to 1.15)
	Intermediate prenatal care (*N* = 126 968)	3107 (2.4)	0.90 (0.87 to 0.94)	0.91 (0.88 to 0.95)
	Adequate prenatal care (*N* = 453 482)	12 475 (2.8)	1 (Ref.)	1 (Ref.)
	Intensive prenatal care (*N* = 344 323)	11 149 (3.2)	1.12 (1.09 to 1.14)	1.03 (1.00 to 1.06)
Severe neonatal morbidity and mortality (SNM‐M)	No prenatal care (*N* = 1833)	201 (11.0)	1.56 (1.36 to 1.78)	1.60 (1.40 to 1.83)
	Inadequate prenatal care (*N* = 28 906)	1765 (6.1)	0.87 (0.83 to 0.92)	0.88 (0.84 to 0.92)
	Intermediate prenatal care (*N* = 126 327)	7135 (5.6)	0.81 (0.79 to 0.83)	0.83 (0.81 to 0.86)
	Adequate prenatal care (*N* = 450 538)	31 477 (7.0)	1 (Ref.)	1 (Ref.)
	Intensive prenatal care (*N* = 343 153)	24 035 (7.0)	1.00 (0.98 to 1.01)	0.90 (0.89 to 0.92)

*Note:* The assessment of SMM‐M was among all livebirths and stillbirths, while the SNM‐M was limited to livebirths.

^a^ For the outcome of SMM‐M, the model was adjusted for maternal age category (≤ 19 [referent], 20–29, 30–39, ≥ 40 years), parity (0 [referent], 1, 2, ≥ 3), rural or urban/missing [referent] residence, neighbourhood income quintile (Q1/missing [referent], Q2, Q3, Q4, Q5), pregnancy or birth as a product of spontaneous ovulation and conception, maternal co‐morbidities categorised by John Hopkins Aggregated Diagnosis Groups (0–2 [referent], 3–4, 5–6, 7–31), 1+ visits to the ED within 365 days before the index pregnancy, and current stillbirth.

^b^ For the outcome of SNM‐M, the model was adjusted for maternal age category (≤ 19 [referent], 20–29, 30–39, ≥ 40 years), parity (0 [referent], 1, 2, ≥ 3), rural or urban/missing [referent] residence, neighbourhood income quintile (Q1/missing [referent], Q2, Q3, Q4, Q5), pregnancy or birth as a product of spontaneous ovulation and conception, maternal co‐morbidities categorised by John Hopkins Aggregated Diagnosis Groups (0–2 [referent], 3–4, 5–6, 7–31), and 1+ visits to the ED within 365 days before the index pregnancy.

Mean values and proportions of baseline variables were contrasted using standardised differences. Modified Poisson regression with generalised estimating equations generated unadjusted and adjusted relative risks (aRR) for SMM‐M and SNM‐M, respectively, comparing the five‐level exposure group of adequacy of prenatal care. The maternal assessment was among all livebirths and stillbirths, while the neonatal assessment was limited to livebirths. Additional analyses were conducted for the outcomes of non‐fatal severe maternal morbidity (SMM) and severe neonatal morbidity (SNM), and also for maternal and neonatal mortality. Analyses were performed using SAS version 9.4 software.

Among 955 814 births, 950 757 (99.5%) were livebirths (Figure S2). A total of 924 773 (96.8%) pregnancies had sufficient prenatal care (mean age 30.9 years), while 31 041 (3.2%) did not (mean age 27.9 years) (Table S3 and Table S4).

SMM‐M occurred among 27 757 (2.9%) women. Relative to receipt of adequate prenatal care, those with no care had an aRR of SMM‐M of 1.42 (95% CI 1.13–1.77) (Table 1). Women with inadequate (aRR 1.08, 95% CI 1.01–1.15) and intensive (aRR 1.04, 95% CI 1.00–1.06) care also had a somewhat higher risk of SMM‐M.

SNM‐M occurred among 64 613 (6.8%) liveborn infants. Compared to newborns of women who received adequate care, those receiving no prenatal care had the greatest aRR of SNM‐M (1.60, 95% CI 1.40–1.83) (Table 1).

The results for non‐fatal SMM and SNM were similar to those seen for SMM‐M and SNM‐M (Table S5). The results for maternal and neonatal mortality followed similar, but more pronounced, patterns (Table S6).

Within a universal healthcare system with complete population‐wide datasets, women without prenatal care and their infants had higher associated risks of severe morbidity and death. A study from Manitoba, Canada, found insufficient prenatal care to be associated with higher odds of stillbirth, preterm birth, small‐for‐gestational‐age birthweight, and admission to the neonatal intensive care unit [5]. The current study demonstrated that women receiving intensive prenatal care had a marginally higher associated rate of morbidity, but their infants did not. The former may be due to confounding by indication, as high‐risk pregnancies are more likely to receive multiple prenatal care visits but may remain vulnerable to maternal complications. Among identified at‐risk women, such as those with pre‐pregnancy comorbid conditions, the association between the intensity of prenatal care and SMM‐M, SNM‐M and other outcomes could be evaluated using a propensity‐score weighted analysis.

Prenatal care using the R‐GINDEX is primarily based on the number of prenatal visits and does not consider the quality of care [2]. Potential confounders, including obesity and smoking status, were unavailable. In addition, we excluded pregnancies ending before 20 weeks, which may be correlated with insufficient prenatal care.

Life‐threatening maternal and newborn morbidity is more apparent among those with little to no prenatal care. Future research and public health programmes should focus on improving access to prenatal care and the quality of that care.

## Author Contributions

Z.L., J.G.R. and N.M.I. contributed to the conception and design of the work. Z.L. and S.S. contributed to the acquisition, analysis and cleaning of data. Z.L. drafted the manuscript. All of the authors revised the manuscript and approved the final version to be published.

## Funding

Z.L. was funded by the Ontario Graduate Scholarship. This research was supported by the Data Sciences Institute at the University of Toronto (DSI‐DAGY3R1P07) and by ICES, which is funded by an annual grant from the Ontario Ministry of Health (MOH) and the Ministry of Long‐Term Care (MLTC). The opinions, results and conclusions reported in this paper are those of the authors and are independent from the funding sources. No endorsement by ICES, the MOH or MLTC is intended or should be inferred. N.M.I. is supported by a Canada Research Chair in Implementation of Evidence‐based Practice and by the Department of Family and Community Medicine at Women’s College Hospital and the University of Toronto.

## Conflicts of Interest

The authors declare no conflicts of interest.

## Supporting information


**Data S1:** bjo70119‐sup‐0001‐Supinfo.docx.

## Data Availability

The dataset from this study is held securely in coded form at ICES. While legal data sharing agreements between ICES and data providers (e.g., healthcare organisations and government) prohibit ICES from making the dataset publicly available, access may be granted to those who meet pre‐specified criteria for confidential access, available at www.ices.on.ca/DAS (email: das@ices.on.ca). The full dataset creation plan and underlying analytic code are available from the authors upon request, understanding that the computer programs may rely upon coding templates or macros that are unique to ICES and are, therefore, either inaccessible or may require modification.
