# Supplementary material for: Adequacy of Prenatal Care and Ensuing Maternal and Neonatal Severe Morbidity and Mortality
Source: BJOG. 2025 Dec 17;133(4):849–51. doi: 10.1111/1471-0528.70119 (PMC12884242; doi:10.1111/1471-0528.70119)
Supplement: Supplementary file 1 — Data S1: bjo70119‐sup‐0001‐Supinfo.docx. [file BJO-133-849-s001.docx]

**Supplemental eFiles. Table of Contents**

[Table S1. List of ICES Databases Used in the Current Study 2](#_Toc213601316)

[Table S2. Cohort Entry and Exclusion Criteria, Methods and Coding to Identify Study Outcomes 3](#_Toc213601317)

[Table S3. World Region of Birth and Corresponding Countries. 22](#_Toc213601319)

[Table S4. Characteristics of all 955,814 singleton hospital births in Ontario according to the adequacy of prenatal care, based on the R-GINDEX. All data are presented as a number (%) unless otherwise indicated. 20](#_Toc213601318)

[Table S5. Adjusted relative risks for the confounders included in the main model presented in Table 2, of the risk of severe maternal morbidity or mortality (SMM-M) within 42 days after the index delivery hospitalization discharge date and severe neonatal morbidity or mortality (SNM-M) within 27 days after the index birth hospitalization discharge date, comparing inadequate vs adequate receipt of prenatal care based on the R-GINDEX. 23](#_Toc213601320)

[Figure S1. Adequate prenatal care and subsequent maternal and neonatal severe morbidity and mortality indicators after birth. Shown is a causal diagram (A) and the timeline for measured variables (B). 25](#_Toc213601321)

[Figure S2. Cohort creation. 26](#_Toc213601322)

## Table S1. List of ICES Databases Used in the Current Study

| **Dataset Name** | **Description** |
| --- | --- |
| Canadian Institute for Health Information Discharge Abstract Database (CIHI-DAD) | Captures all in-patient hospital admission records including obstetric deliveries and deaths. Diagnostic codes are based on the International Statistical Classification of Diseases and Related Health Problems, Tenth Revision, Canada (ICD-10-CA), and procedural codes are based on the Canadian Classification of Health Interventions (CCI). |
| National Ambulatory Care Reporting System (NACRS) | Contains administrative, clinical, demographic, and administrative information for all patient visits made to hospital and community-based ambulatory care centres. |
| Ontario Health Insurance Plan Claims Database (OHIP) | Contains information on inpatient and outpatient services provided to Ontario residents eligible for the province’s publicly funded health insurance system by fee for-service health care practitioners and “shadow billings” for those paid through non-fee-for-service payment plans. The main data elements include patient and physician identifiers, code for service provided,  date of service, associated diagnosis, and fee paid. |
| Same Day Surgery Database (SDS) | Contains administrative, clinical, demographic, and administrative information for all patient visits made to day surgery institutions  in Ontario. The main data elements include  patient demographics, clinical data, administrative data, financial data, service-specific data elements for day surgery and emergency. |
| Linked Delivering Mothers and Newborns (MOMBABY) | Derived from CIHI-DAD, provides linked inpatient hospital admission records of mothers and their infants. |
| Better Outcomes Registry and Network (BORN)  BORN Information System (BIS) | Ontario’s perinatal, newborn, and child registry. BIS is a database established to collect, manage, protect and share critical data about every pregnancy, birth and child in Ontario. |
| Registered Persons Database (RPDB) | Includes vital status and sociodemographic information about all individuals who have ever received an Ontario Health Insurance Plan (OHIP) number (e.g., date of birth, sex, and postal code). |
| Postal Code Conversion File Plus (PCCF+) | A digital file that links the Canada Post Corporation (CPC) six-character postal code and Statistics Canada’s standard geographic areas (e.g., dissemination area). Area-level income quintiles ranges from Q1 (lowest) to Q5 (highest) income neighbourhoods. |
| Immigration, Refugees and Citizenship Canada Permanent Residents database (IRCC-PR) | Contains demographic information on all international migrants who obtained permanent residency in Canada from January 1985 to May 2017. |

## Table S2. Cohort Entry and Exclusion Criteria, Methods and Coding to Identify Study Outcomes

| **Assessment** | **Timing** | **Disease, procedure or condition** | **ICD-10-CA or CCI codes in CIHI-DAD and NACRS** | **Diagnostic & fee codes in OHIP** | **Other Sources** |
| --- | --- | --- | --- | --- | --- |
| **INCLUSION CRITERIA** | April 1, 2012 to March 31, 2020, at the time of the mother's index delivery hospitalization date (admission date to the episode of care for the birth) | All hospital-based singleton livebirths or stillbirths at 20 to 42 weeks’ gestation, in Ontario, Canada | MOMBABY (links the hospital admission records of delivering mothers and newborns in CIHI-DAD) | --- | -- |
| **EXCLUSION CRITERIA** | At the time of index delivery hospitalization | Females who were a non-Ontario resident | --- | --- | RPDB:  (substr(prcddablk,1,2) ne ‘35’) |
|  | At the time of index delivery hospitalization | Females who had an invalid OHIP number  or hospital number & infants who had an invalid OHIP number | MOMBABY/RPDB: Invalid M_IKN  a. VALIKN ne ‘V’  b. M_IKN not in RPDB (according to no  sex & no bdate)  c. M_IKN with sex =’M’ in RPDB | --- | --- |
|  | At the time of index delivery hospitalization | Females ineligible for OHIP | --- | --- | RPDB |
|  | At the time of index delivery hospitalization | Records with warning for mother’s or infants’ IKN or KEY | MOMBABY/RPBD: warning for IKN/KEY (WARN not =”N”(No Warning)). Include N=no warning | --- | --- |
|  | At the time of index (infant) birth admission | Infant gestational age is out of range | MOMBABY  a. B_GESTWKS_DEL  b. gestational age <20 or ≥43 weeks | --- | BORN-BIS (2012-2018):  a. GA_AT_BIRTH_WEEKS  b. gestational age <20 or ≥43 weeks |
| **MAIN EXPOSURES**  **Maternal exposure:** Receipt of adequate prenatal care: R-GINDEX | i, ii, iii) During the index pregnancy  iv) At the time of index (infant) birth admission | i) Occurrence of prenatal visit with a midwife, family physician and/or obstetrician, in the first trimester (0-12 weeks gestation)  AND  ii) Total number of prenatal visits  AND  Iii) Mom’s gestational weeks at admission and delivery  AND  iV) Infant gestational age at birth.  These will be used to calculate the R-GINDEX for a composite measure of receipt of adequate prenatal care on a 5-level exposure: no care, inadequate, intermediate, adequate, intensive. | MOMBABY:  M_GESTWKS_ADM  M_GESTWKS_DEL B_GESTWKS_DEL | Family Physician:  A007, A001, A003, K013  Obstetrician:  P002, A205, A203, A206, A204  Fam Phys/Obs:  P003, P004, P005 | Alexander & Kotelchuck. Quantifying the adequacy of prenatal care: a comparison of indices. Public Health Rep. 1996.  BORN-BIS (2012-2018):  GA_AT_BIRTH_WEEKS  MW_FIRSTTRIMVISITPROVIDER_ID  a. 1025430 = Midwife  b. 1025440 = Midwife and other  MWPRENNUMOFVISITSCOORDMW  MWPRENNUMOFVISITSOTHERMW  MW_BILLABILITY_TYPE_ID  GA_AT_BIRTH_WEEKS |
| **OUTCOMES**  **Maternal outcome:**  Any severe maternal morbidity and mortality (SMM-M) indicators among women with a livebirth or stillbirth | From the mother’s index delivery hospitalization and up to 42 days thereafter | SMM-M: 0 or 1+ SMM-M indicators | Severe preeclampsia and (HELLP) syndrome: O14.1, or O14.2  Eclampsia: O15  Cerebral venous thrombosis in pregnancy, or in the puerperium: O22.5, or O87.3  Acute fatty liver with red blood cell (RBC) transfusion or plasma transfusion: O26.6 + (CIHI BTREDBC = 1 or CIHI BTPLASMA = 1)  Pulmonary, cardiac, and CNS complications of anesthesia during pregnancy, the puerperium or labour and delivery: O29.0, O29.1, O29.2, O89.0, O89.1, O89.2, O74.0, O74.1, O74.2 or O74.3  Placenta previa with hemorrhage with RBC transfusion: O44.1 + CIHI BTREDBC = 1  Placental abruption with coagulation defect: O45.0  Antepartum hemorrhage with coagulation defect: O46.0  Intrapartum hemorrhage with coagulation defect: O67.0  Intrapartum hemorrhage with RBC transfusion: O67 + CIHI BTREDBC = 1  Rupture of the uterus with RBC transfusion, procedures to the uterus or hysterectomy: (O71.0 or O71.1) + any of the following:   - CIHI BTREDBC = 1, or - (1.RM.13, 1.KT.51, 5.PC.91.LA or 5.PC.91.HV) + CIHI BTREDBC = 1, or - (5.MD.60.RC, 5.MD.60.RD, 5.MD.60.KE, 5.MD.60.CB or 1.RM.89.LA^a^ ), or - 1.RM.87.LA-GX   **^a^NOTE**: **1.RM.89.LA** is included only if codes 1.PL.74, 1.RS.74 or 1.RS.80 are NOT also present  Postpartum hemorrhage with RBC transfusion, procedures to the uterus or hysterectomy: O72 + any of the following:   - BTREDBC = 1, or - (1.RM.13, 1.KT.51, 5.PC.91.LA or 5.PC.91.HV) + BTREDBC = 1, or - (5.MD.60.RC, 5.MD.60.RD, 5.MD.60.KE, 5.MD.60.CB or 1.RM.89.LA^b^ ), or - 1.RM.87.LA-GX   **^b^NOTE:** 1.RM.89.LA is included only if codes 1.PL.74, 1.RS.74 or 1.RS.80 are NOT also present  Cardiac conditions: O74.2, O89.1, O90.3, I21, I22, I42, I43, I46, I49.0, I50, J81, 1.HZ.09 or 1.HZ.30  Obstetric shock: O75.1, R57, T80.5 or T88.6  Septicemia during labour: O75.3  Complications of obstetric surgery and procedures: O75.4  Puerperal sepsis: O85  Obstetric embolism: O88  Acute renal failure: O90.4, N17, N19 or N99.0  Disseminated intravascular coagulation: D65  Sickle cell anemia with crisis: D57.0  Acute psychosis: F53.1 or F23  Status epilepticus: G41  Cerebral edema or coma: G93.6 or R40.2  Cerebrovascular diseases: subarachnoid and intracranial hemorrhage, cerebral infarction, stroke: I60, I61, I62, I63 or I64  Status asthmaticus: J45.01, J45.11, J45.81 or J45.91  Adult respiratory distress syndrome: J80  Acute abdomen: K35, K37, K65, N73.3 or N73.5  Hepatic failure: K71 or K72  Assisted ventilation through endotracheal tube: 1.GZ.31.CA-ND  Assisted ventilation through tracheostomy: 1.GZ.31.CR-ND  Hysterectomy: 5.MD.60.RC, 5.MD.60.RD, 5.MD.60.KE, 5.MD.60.CB, 1.RM.89.LA (exclude if 1.PL.74, 1.RS.74 or 1.RS.80 code also present), 1.RM.87.LA-GX  Dialysis: 1.PZ.21  Evacuation of incisional hematoma with RBC transfusion: 5.PC.73.JS + CIHI BTREDBC = 1  Repair of bladder, urethra, or intestine: 5.PC.80.JR, 1.NK.80, 1.NM.80  Procedures to the uterus/pelvic vessels with RBC transfusion: (1.RM.13, 1.KT.51, 5.PC.91.LA, 5.PC.91.HV) + CIHI BTREDBC = 1  Surgical or manual correction of inverted uterus for vaginal births only: 5.PC.91.HQ or 5.PC.91.HP, restricted to vaginal births (i.e., absence of caesarean 5.MD.60)  Reclosure of caesarean wound with RBC transfusion: (5.PC.80.JM, 5.PC.80.JH) + CIHI BTREDBC = 1  Curettage with RBC transfusion: (5.PC.91.GA, 5.PC.91.GC, 5.PC.91.GD) + CIHI BTREDBC = 1  Maternal ICU admission: SCU in (‘10’, ’20’, ’25’, ’30’, ’35’, ’40’,’45’,’60’,’80’)  Maternal Death (captured in CIHI-DAD or RPDB) |  | Dzakpasu S, et al. Severe maternal morbidity surveillance: Monitoring pregnant women at high risk for prolonged hospitalisation and death. Paediatr Perinat Epidemiol. 2020. |
| **Infant outcome:**  Number of severe neonatal morbidity and mortality (SNM-M) indicators among liveborn infants | From the infant’s index birth hospitalization and up to 27 days thereafter. | SNM-M: 0 or 1+ SNM-M indicators | Gestational age < 32 weeks MOMBABY: B_GESTWKS_DEL  Birthweight < 1500 grams MOMBABY: B_WEIGHT  Respiratory distress syndrome P22.0  Seizures  P90, R56  Intraventricular haemorrhage (grades 2, 3 or 4) P52.1, P52.2  Cerebral infarction  I63  Periventricular leukomalacia  P91.2    Birth Trauma  P10.0-3, P13.0, P13.2-3, P14.0-4, P14.8-9    Hypoxic ischemic neonatal encephalopathy  P91.5, P91.8, P91.6   Necrotising enterocolitis  P77   Sepsis/septicaemia Infection (streptococcus staphylococcus, E.coli, unspecified gram negative)  P36 G00-03, G05, A40, A41.5, A41.8, A41.9, B95.1, B96.2    Pneumonia  P23; J12-18    Other respiratory (primary atelectasis, respiratory failure)  P28.0; P28.5    Chronic respiratory disease originating in the perinatal period  P27  Perinatal intestinal perforation  P78.0  Retinopathy of prematurity  H35.1  Transfusion of red blood cells or a blood product  1LZ19HHU1A, 1LZ19HHU1J, 1LZ19HHU2A, 1LZ19HHU2J, 1LZ19HHU3J, 1LZ19HHU4J, 1LZ19HHU5J, 1LZ19HHU6A, 1LZ19HHU6J, 1LZ19HHU9A, 1LZ19HHU9J, 1LZ19HMU1, 1LZ19HMU2, 1LZ19HMU9, 1LZ35HAC5  Resuscitation by intubation and/or chest compressions 1HZ30JN, 1HZ30JY, 1GZ30CJ, 1GZ30CJNB, 1GZ30JH    Ventilatory support (mechanical ventilation and/or CPAP)  1GZ31CAEP, 1GZ31CAND, 1GZ31CAPK, 1GZ31CBND, 1GZ31CRND, 1GZ31GPND, 1GZ31JAMD, 1GZ31JANC, 1GZ31JAPK  Central venous or arterial catheter  1KV53HACH, 1KV53HAFT, 1KV53LAFT, 2IM28GP, 2LZ28GQPL, 2LZ28GRPL, 2LZ28JAPL, 1KX53HACH, 1KX53HAFT, 1KX53LAFT, 2LZ28GQPL, 2LZ28GRP, 1IS53^^ Up until 2015 umbilical venous catheterization (UVC) was classified in these codes. After 2015, they were found in 1IS53^^.    Pneumothorax requiring intercostal catheter  1GV52DA, 1GV52DATS, 1GV52HA, 1GV52HAHE, 1GV52HATK, 1GV52LA, 1GV52LATS, 1GV52LAXXE, 1GV54JATS, 1GV55JATS  Any intravenous fluids  1LZ35CAE6, 1LZ35HAC1, 1LZ35HAC5,  1LZ35HAC6, 1LZ35HAC7, 1LZ35HAE6,  1LZ35HAT7, 1LZ35HAT9, 1LZ35HAZ9, 1LZ35HHC1, 1LZ35HHC5, 1LZ35HHC6, 1LZ35HHC7, 1LZ35HHE0, 1LZ35HHE6, 1LZ35HHT7, 1LZ35HHT9, 1LZ35HHZ9, 1LZ35HRC5, 1LZ35HRC6, 1LZ35HRC7, 1LZ35HRT9, 1LZ35HRZ9    Any body cavity surgical procedure  1AA52, 1AA87, 1AC87, 1AE87, 1AF87, 1AG87, 1AJ87, 1AK87, 1AN52, 1AN59, 1AN87, 1AP59, 1AP72, 1AP87, 1AW59, 1AW72, 1AW87, 1AX87, 1BA72, 1BA80, 1BA87, 1BB72, 1BB80, 1BB87, 1BD72, 1BD80, 1BD87, 1BF80, 1BG72, 1BG80, 1BG87, 1BK59, 1BM72, 1BM80, 1BM87, 1BN72, 1BN80, 1BN87, 1BP72, 1BP80, 1BP87, 1BQ72, 1BQ80, 1BQ87, 1BS72, 1BS80, 1BS87, 1BT72, 1BT80, 1BT87, 1GA87, 1GA89, 1GB87, 1GB89, 1GD89, 1GE80, 1GE87, 1GE89, 1GE91, 1GH84, 1GJ86, 1GJ87, 1GK87, 1GK89, 1GM80, 1GM86, 1GM87, 1GN92, 1GR87, 1GR89, 1GR91, 1GT78, 1GT87, 1GT89, 1GT91, 1GV87, 1GV89, 1GW87, 1GX80, 1GX86, 1GX87, 1GY70, 1GY72, 1GY86, 1HJ76, 1HJ82, 1HN87, 1HP76, 1HP78, 1HP80, 1HP82, 1HP83, 1HP87, 1HR80, 1HR84, 1HR87, 1HS80 (excl. 1HS80G), 1HS90, 1HT80 (excl. 1HT80G), 1HT89, 1HT90, 1HU80 (excl. 1HU80G), 1HU90, 1HV80 (excl. 1HV80G), 1HV90, 1HW78, 1HW79, 1HX80, 1HX87, 1HX80, 1HZ87, 1IA76, 1IA80, 1IA87, 1IB76, 1IB79, 1IB80, 1IB82, 1IB87, 1IC76, 1IC80, 1IC82, 1IC87, 1ID76, 1ID80, 1ID82, 1ID86, 1ID87, 1IF83, 1IJ76, 1IJ80, 1IM76, 1IM80, 1IM82, 1IM83, 1IM87, 1IN83, 1IN84, 1IN87, 1JE57 (excl. 1JE57G), 1JE76, 1JE80, 1JE87, 1JJ76, 1JJ80, 1JK76, 1JK80, 1JK87, 1JW51 (excL. 1JW51G), 1JW57, 1JW76, 1LA84, 1LC84, 1LD84, 1NA72, 1NA74, 1NA76, 1NA77, 1NA80, 1NA84, 1NA86, 1NA87, 1NA88, 1NA89, 1NA90, 1NA91, 1NA92, 1NE80, 1NF76, 1NF78, 1NF80, 1NF82, 1NF84, 1NF86, 1NF87 (excl. 1NF87B), 1NF89, 1NF90, 1NF91, 1NF92, 1NK76, 1NK77, 1NK80, 1NK82, 1NK84, 1NK87 (excl. 1NK87B), 1NM74, 1NM76, 1NM77, 1NM80, 1NM82, 1NM87 (excl. 1NM87B), 1NM89, 1NM91, 1NP72, 1NP73, 1NP86, 1NQ74 (excl. 1NQ74B), 1NQ80, 1NQ84, 1NQ86, 1NQ87 (excl. 1NQ87B), 1NQ89, 1NQ90, 1NT80, 1NT84, 1NT86, 1NT87, 1NV89, 1OA87, 1OB87, 1OB89, 1OD76, 1OD89, 1OE76, 1OE80, 1OE89, 1OJ76 (excl. 1OJ76B), 1OJ87, 1OJ89, 1OK87, 1OK89, 1OK91, 1OT72, 1OT87, 1OT91, 1PB87, 1PB89, 1PC80, 1PC87 (excl. 1PC87D), 1PC89, 1PC91, 1PE57 (excl. 1PE57BD), 1PE80 (excl. 1PE80D), 1PE82, 1PE87 (excl. 1PE87D), 1PE89 (excl. 1PE89D), 1PG76, 1PG77, 1PG80 (excl. 1PG80D), 1PG86, 1PG89, 1PL74 (excl. 1PL74CD), 1PL80, 1PM79, 1PM86, 1PM87 (excl. 1PM87B), 1PM89, 1PM90, 1PM91, 1PM92, 1QE53, 1QE80, 1QE82, 1QE84, 1QE87, 1QE89, 1QG89, 1QM74, 1QM80, 1QM87, 1QM89, 1QM91, 1QN82, 1QT87, 1QT91, 1RB74, 1RB80, 1RB83, 1RB87, 1RB89,1RD89, 1RF51, 1RF72, 1RF74, 1RF80, 1RF87, 1RF89, 1RM87 (excl. 1RM87B), 1RM89, 1RM91, 1RN87, 1RN89, 1RS74, 1RS80, 1RS86, 1RS87, 1RS89, 1RW87, 1RW88, 1RW91, 1RW92, 1SA74, 1SA75, 1SA80, 1SA89, 1SC74, 1SC75, 1SC80, 1SC87, 1SC89, 1SE53, 1SE89 (excl. 1SE89D), 1SF80, 1SF87, 1SF89, 1SG80, 1SG87, 1SH87, 1SM74, 1SM80, 1SM87, 1SN87, 1SN93, 1SQ53, 1SQ74, 1SQ80, 1SQ87, 1SQ91, 1SQ93, 1SW74, 1SY80, 1SY84, 1SY87, 1SZ87, 1VA53, 1VA74, 1VA75, 1VA80, 1VA87, 1VA93, 1VC74, 1VC80, 1VC87, 1VC91, 1VC93, 1VE80, 1VG53, 1VG55, 1VG72, 1VG73, 1VG74, 1VG75, 1VG80, 1VG87, 1VG93, 1VK80, 1VK87, 1VK89, 1VL80, 1VL87, 1VM80, 1VM87, 1VN80, 1VN87, 1VP74, 1VP80, 1VP87, 1VP89, 1VQ74, 1VQ79, 1VQ80, 1VQ82, 1VQ87, 1VQ91, 1VQ93, 1VS72, 1VS80, 1VX87 | Hypothermia (therapeutic) induction G210 | Nelson CRM, et al. Neonatal Adverse Outcomes among Hospital Livebirths in Canada: A National Retrospective Study. Neonatology. 2024. |
| **BASELINE CHARACTERISTICS** | At the time of index delivery hospitalization | Rural or urban residence: Mother's postal code will be used to derive rural or urban residence | --- | --- | RPDB:  a. PSTLCODE  PCCF+/Statistics Canada Census Data |
|  | At the time of mother’s arrival to Canada | World Region of Birth: Canada, Western  Nations & Europe, Hispanic America,  Caribbean, Sub-Saharan Africa, Middle East &  North Africa, East Asia & Pacific, South Asia (See eTable 3) | --- | --- | IRCC-PRD: COUNTRY_BIRTH |
|  | Mother's landing date in Canada & date of index (infant) birth admission | Duration of residence in ON: subtracted landing date from mother's index delivery hospitalization date (baby’s date of birth) divided by 365.25 | MOMBABY:  B_BDATE | --- | IRCC-PRD:  LANDING_DATE |
|  | At the time of index delivery hospitalization | Maternal Primary Language |  | --- | BORN-BIS (2018-2021):  PRIMARY_LANGUAGE_ID |
|  | At the time of index delivery hospitalization | Mother’s postal code to derive neighbourhood income quintile, ranging from Q1 (lowest) to Q5 (highest) | --- | --- | RPDB:  a. PSTLCODE  PCCF+/Statistics Canada Census Data: |
|  | At the time of the index delivery hospitalization on the maternal record | Delivery by caesarean (yes/no) | Caesarean section: CCI code 5MD60 | --- | --- |
|  | At the time of the index delivery hospitalization | Perineal laceration injury during delivery | Third degree: O70.2  Fourth degree: O70.3 | --- | --- |
| **Confounders** | At the time of the index delivery hospitalization, admission date for the birth episode of care | Maternal age (years) | --- | --- | RPDB |
|  | At the time of the index delivery hospitalization | Parity; number of times an individual has given birth | MOMBABY  a. M_PREVBIRTH_DERIVED  b. M_PREVBIRTH_ORIGINAL | --- | --- |
|  | Within 365 days before the estimated clinical start of pregnancy (i.e. 0 weeks’ gestation) | Total number of John Hopkins Aggregated Diagnosis Groups (ADGS; 0 to 2, 3 to 4, 5 to 6, 7 to 31) | Diagnosis codes in DAD, SDS, OHIP, and NACRS using ACG system software  Exclude any pregnancy ADGs | --- | --- |
|  | At the time of index delivery hospitalization and (infant) birth admission | Pregnancy or birth was a product of assisted reproductive technology (ART) or spontaneous ovulation & conception | MOMBABY  a. M_PRODUCT  b. ART=A  c. Spontaneous=N | --- | BORN-ACG:  CONCEPTION_TYPE_ID  ART = 1013130, 1013140, 1013110, 1013120, 1013150, 3000006  Spontaneous = 1013160 |
|  | Within 365 days before the estimated clinical start of pregnancy (i.e. 0 weeks’ gestation) | Number of maternal ED visits (count) | Any ICD-10-CA code in NACRS | --- | --- |
|  | Within 365 days before the estimated clinical start of pregnancy (i.e. 0 weeks’ gestation) | Occurrence of 0 or 1+ visits with an FP or GP |  | Family Physician or General Practitioner:  A001, A003, A004, A005, A006, A007, A008, A901, A903, A905, G212, G271, G372, G373, G365, G538, G539, G590, G591, G842, G843, G844, G845, G846, G847, G848, K005, K013, K017 |  |

Abbreviations: ICD-10-CA: International Classification of Diseases, 10th Revision, Canada; CCI: Canadian Classification of Health Interventions; CIHI: Canadian Institute for Health Information; DAD: Discharge Abstract Database; NACRS: National Ambulatory Care Reporting System; OHIP: Ontario Health Insurance Plan Claims Database; MOMBABY: Linked Delivering Mother and Newborns; RPDB: Registered Persons Database; Better Outcomes Registry and Network (BORN); BORN Information System (BIS); PCCF+: Postal code conversion file plus (Statistics Canada); IRCC-PRD: Immigration, Refugees, and Citizenship Canada Permanent Resident Database; SDS: Same Day Surgery Database.

##

## Table S3. World Region of Birth and Corresponding Countries.

| **World Region** | **Countries^a^** |
| --- | --- |
| **Western nations and Europe** | Albania, Andorra, Australia, Austria, Belarus, Belgium, Bermuda, Bosnia and Herzegovina, Bulgaria, Croatia, Czech Republic, Denmark, Estonia, Finland, Former Czechoslovakia, Former USSR, Former Yugoslavia, France, Germany, Greece, Greenland, Hungary, Iceland, Ireland, Italy, Kosovo, Latvia, Liechtenstein, Lithuania, Luxembourg, Macedonia, Malta, Moldova, Monaco, Montenegro, Netherlands, New Zealand, Norway, Poland, Portugal, Romania, Russian Federation, San Marino, Serbia, Serbia and Montenegro, Slovakia, Slovenia, Spain, St Pierre and Miquelon, Sweden, Switzerland, Ukraine, United Kingdom, United States, Vatican City State |
| **Sub-Saharan Africa** | Angola, Benin, Botswana, Burkina Faso, Burundi, Cameroon, Cape Verde, Central African Republic, Chad, Comoros, Congo, The Democratic Republic of Congo, Cote D'Ivoire, Djibouti, Equatorial Guinea, Eritrea, Ethiopia, Gabon, Gambia, Ghana, Guinea, Guinea Bissau, Kenya,  Lesotho, Liberia, Madagascar, Malawi, Mali, Mauritania, Mauritius, Mayotte, Mozambique, Namibia, Niger, Nigeria, Reunion, Rwanda, Sao Tome and Principe, Senegal, Seychelles, Sierra Leone, Somalia, South Africa, Swaziland, Tanzania, United Republic Of Togo, Uganda, Western Sahara, Zambia, Zimbabwe |
| **The Caribbean** | Anguilla, Antigua and Barbuda, Aruba, Bahamas, Barbados, Belize, Cayman Islands, Dominica, Grenada, Guadeloupe, Haiti, Jamaica, Martinique, Montserrat, Netherlands Antilles, Saint Kitts and Nevis, Saint Lucia, Saint Vincent And The Grenadines, Trinidad and Tobago, Turks and Caicos Islands, Virgin Islands, British Virgin Islands |
| **North Africa and the Middle East** | Algeria, Armenia, Azerbaijan, Bahrain, Cyprus, Egypt, Georgia, Iran,  Iraq, Israel, Jordan, Kazakhstan, Kuwait, Kyrgyzstan, Lebanon, Libya, Morocco, Oman, Occupied  Palestinian Territory, Qatar, Saudi Arabia, Sudan, Syria, Tajikistan, Tunisia, Turkey, Turkmenistan, United  Arab Emirates, Uzbekistan, Yemen |
| **Hispanic America** | Argentina, Bolivia, Brazil, Chile, Colombia, Costa Rica, Cuba, Dominican Republic,  Ecuador, El Salvador, French Guiana, Guatemala, Guyana, Honduras, Mexico, Nicaragua, Panama,  Paraguay, Peru, Puerto Rico, Suriname, Uruguay, Venezuela |
| **East Asia and Pacific** | Brunei Darussalam, Cambodia, China, Fiji, French Polynesia, Guam, Hong Kong,  Indonesia, Japan, Kiribati, Korea North, Korea South, Laos, Macau, Malaysia, Mongolia, Myanmar, Nauru,  New Caledonia, Northern Mariana Islands, Palau, Papua New Guinea, Philippines, Samoa, Singapore,  Solomon Islands, Taiwan, Thailand, Tibet, Tonga, Vanuatu, Viet Nam, Tuvalu, Marinas, Republic of The  Marshall Islands, Federated States of Micronesia, Cook Islands, Wallis and Futuna, East Timor |
| **South Asia** | Afghanistan, Bangladesh, Bhutan, India, Maldives, Nepal, Pakistan, Sri Lanka |

^a^The list reflects the names of the maternal countries of birth at the time of their immigration to Canada, and therefore, may include country names that no longer exist.

## Table S4. Characteristics of all 955,814 singleton hospital births in Ontario according to the adequacy of prenatal care, based on the R-GINDEX. All data are presented as a number (%) unless otherwise indicated.

| **Characteristic** | **Sufficient**  **prenatal care**  **(N = 924,773)^a^** | **Insufficient**  **prenatal care**  **(N = 31,041)^b^** | **Standardized difference^c^** |
| --- | --- | --- | --- |
| **Of the mother** |  |  |  |
| Age at index birth, Mean ± SD, y | 30.9 ± 5.2 | 27.9 ± 6.0 | 0.53 |
| *≤ 19* | 18,126 (2.0) | 2,472 (8.0) | 0.04 |
| *20-29* | 332,534 (36.0) | 16,315 (52.6) | 0.34 |
| *30-39* | 532,851 (57.6) | 11,392 (36.7) | 0.43 |
| *≥ 40* | 41,262 (4.5) | 862 (2.8) | 0.09 |
| Parity category |  |  |  |
| *0* | 398,779 (43.1) | 10,379 (33.4) | 0.20 |
| *1* | 340,399 (36.8) | 9,680 (31.2) | 0.12 |
| *2* | 125,326 (13.6) | 5,388 (17.4) | 0.11 |
| *≥ 3* | 60,269 (6.5) | 5,594 (18.0) | 0.36 |
| Rural Residence |  |  |  |
| *Rural* | 88,487 (9.6) | 9,602 (30.9) | 0.55 |
| *Urban* | 835,163 (90.3) | 21,362 (68.8) | 0.55 |
| *Missing* | 1,123 (0.1) | 77 (0.2) | 0.03 |
| World Region of Birth |  |  |  |
| *Canada^b^* | 728,382 (78.8) | 26,286 (84.7) | 0.15 |
| *East Asia & Pacific* | 52,707 (5.7) | 835 (2.7) | 0.15 |
| *Hispanic America* | 12,988 (1.4) | 325 (1.0) | 0.03 |
| *North Africa & Middle East* | 21,785 (2.4) | 1,114 (3.6) | 0.07 |
| *South Asia* | 63,442 (6.9) | 1,078 (3.5) | 0.15 |
| *Sub-Saharan Africa* | 14,186 (1.5) | 659 (2.1) | 0.04 |
| *The Caribbean* | 7,699 (0.8) | 230 (0.7) | 0.01 |
| *Western Nations & Europe* | 23,584 (2.6) | 514 (1.7) | 0.06 |
| Duration of residence in Ontario preceding the index birth, Mean ± SD, y | 6.8 ± 4.4 | 6.9 ± 4.6 | 0.03 |
| *< 5* | 75,126 (8.1) | 1,719 (5.5) | 0.11 |
| *5-9* | 57,473 (6.2) | 1,343 (4.3) | 0.08 |
| *≥ 10* | 46,780 (5.1) | 1,228 (4.0) | 0.05 |
| *Missing* | 745,394 (80.6) | 26,751 (86.2) | 0.03 |
| Primary language |  |  |  |
| *English* | 720,474 (77.9) | 23,900 (77.0) | 0.02 |
| *French* | 12,694 (1.4) | 382 (1.2) | 0.01 |
| *Other* | 68,335 (7.4) | 2,765 (8.9) | 0.06 |
| *Missing* | 123,270 (13.3) | 3,994 (12.9) | 0.01 |
| Neighbourhood Income Quintile (Q) |  |  |  |
| *Q1 (lowest)* | 192,133 (20.8) | 11,290 (36.4) | 0.35 |
| *Q2* | 183,391 (19.8) | 6,220 (20.0) | 0.01 |
| *Q3* | 193,736 (20.9) | 5,264 (17.0) | 0.10 |
| *Q4* | 196,540 (21.3) | 4,448 (14.3) | 0.18 |
| *Q5 (highest)* | 156,756 (17.0) | 3,337 (10.8) | 0.18 |
| *Missing* | 2,217 (0.2) | 482 (1.6) | 0.14 |
| Johns Hopkins Aggregated Diagnosis Groups |  |  |  |
| *0 to 2* | 374,278 (40.5) | 19,706 (63.5) | 0.47 |
| *3 to 4* | 272,553 (29.5) | 6,407 (20.6) | 0.20 |
| *5 to 6* | 164,176 (17.8) | 3,069 (9.9) | 0.23 |
| *7 to 31* | 113,766 (12.3) | 1,859 (6.0) | 0.22 |
| Pregnancy is a product of spontaneous ovulation & conception | 889,639 (96.2) | 30,795 (99.2) | 0.20 |
| Caesarean Delivery | 261,743 (28.3) | 7,045 (22.7) | 0.13 |
| Gestational Age at index birth, Mean ± SD, weeks | 38.8 ± 2.0 | 38.8 ± 1.9 | 0.00 |
| Severe perineal laceration during the delivery |  |  |  |
| *Third degree* | 22,389 (2.4) | 507 (1.6) | 0.06 |
| *Fourth degree* | 2,567 (0.3) | 49 (0.2) | 0.03 |
| Number of ED visits within 365 d before the index pregnancy, Mean ± SD | 0.6 ± 1.5 | 0.9 ± 1.8 | 0.14 |
| Had 1+ visits to the ED within 365 d before the index pregnancy | 284,185 (30.7) | 11,180 (36.0) | 0.11 |
| Number of visits to a primary care physician before the index pregnancy, Mean ± SD | 3.5 ± 3.8 | 1.28 ± 2.3 | 0.72 |
| Had 1+ visits to a primary care physician before the index pregnancy | 776,582 (84.0) | 14,829 (47.8) | 0.83 |
| Type of healthcare professional |  |  |  |
| *Obstetrician* | 663,205 (71.7%) | 17,568 (56.6%) | 0.32 |
| *Family Physician* | 34,710 (25.4%) | 12,355 (39.8%) | 0.31 |
| *Midwife* | 125,792 (13.6%) | 2,164 (7.0%) | 0.22 |
| *Other^b^* | 69,814 (7.5%) | 5,383 (17.3%) | 0.30 |
| *None* | 1,952 (0.2%) | 1,548 (5.0%) | 0.30 |
| **Of the newborn** |  |  |  |
| Female sex^e^ | 450,312 (48.7) | 15,082 (48.6) | 0.00 |
| Birthweight^e^, Mean ± SD, grams | 3359 ± 565 | 3346 ± 573 | 0.02 |
| Preterm birth < 37 weeks’ gestation^e^ | 60,995 (6.6) | 2,333 (7.5) | 0.04 |
| Livebirth | 920,018 (99.5) | 30,739 (99.0) | 0.05 |
| Stillbirth | 4,755 (0.5) | 302 (1.0) | 0.05 |

SD: standard deviation; ART: assisted reproductive technology; ED: emergency department.

^a^Sufficient prenatal care comprises adequate prenatal care or intensive prenatal care, according to the R-GINDEX.

^b^Inusfficient prenatal care comprises no prenatal care, inadequate prenatal care or intermediate prenatal care, according to the R-GINDEX.

^c^A standardized difference of greater than 0.10 is considered to be clinically meaningful.

^d^Includes corresponding missing category for World region of birth, and type of healthcare professional.

^e^Includes livebirths only.

Table S5. Risk of severe maternal morbidity (SMM) without maternal death within 42 days after the index delivery hospitalization discharge date (upper), and severe neonatal morbidity (SNM) without neonatal death within 27 days after the index birth hospitalization discharge date (lower), each by adequacy of prenatal care based on the R-GINDEX. The assessment of SMM was among all livebirths and stillbirths, while the SNM outcome was limited to livebirths.

|  |  |  | **Relative Risk (95% CI)** | |
| --- | --- | --- | --- | --- |
| **Outcome** | **Exposure group** | **No. (%) with outcome** | **Unadjusted** | **Adjusted^a, b^** |
| **SMM without death** | No prenatal care  (N = 1923) | 96 (5.0) | 1.51 (1.20 to 1.90) | 1.41 (1.12 to 1.77) |
|  | Inadequate prenatal care  (N = 29,118) | 926 (3.2) | 1.08 (1.01 to 1.15) | 1.06 (0.99 to 1.14) |
|  | Intermediate prenatal care  (N = 126,968) | 3102 (2.4) | 0.90 (0.87 to 0.94) | 0.91 (0.88 to 0.94) |
|  | Adequate prenatal care  (N = 453,482) | 12,466 (2.7) | 1 (Ref.) | 1 (Ref.) |
|  | Intensive prenatal care  (N = 344,323) | 11,143 (3.2) | 1.12 (1.09 to 1.14) | 1.04 (1.02 to 1.07) |
|  |  |  |  |  |
| **SNM without death** | No prenatal care  (N = 1833) | 200 (10.9) | 1.55 (1.36 to 1.78) | 1.62 (1.42 to 1.86) |
|  | Inadequate prenatal care  (N = 28,906) | 1751 (6.1) | 0.87 (0.83 to 0.91) | 0.88 (0.83 to 0.92) |
|  | Intermediate prenatal care  (N = 126,327) | 7101 (5.6) | 0.81 (0.79 to 0.83) | 0.83 (0.81 to 0.85) |
|  | Adequate prenatal care  (N = 450,538) | 31418 (7.0) | 1 (Ref.) | 1 (Ref.) |
|  | Intensive prenatal care  (N = 343,153) | 23987 (7.0) | 1.00 (0.98 to 1.01) | 0.91 (0.89 to 0.92) |

^a^For the outcome of SMM, the model was adjusted for maternal age category (≤ 19 [referent], 20-29, 30-39, ≥ 40 y), parity (0 [referent], 1, 2, ≥ 3), rural or urban/missing [referent] residence, neighbourhood income quintile (Q1/missing [referent], Q2, Q3, Q4, Q5), pregnancy or birth as a product of spontaneous ovulation & conception, maternal co-morbidities categorized by John Hopkins Aggregated Diagnosis Groups (0-2 [referent], 3-4, 5-31), 1+ visits to the ED within 365 d before the index pregnancy, and current stillbirth.

^b^For the outcome of SNM, the model was adjusted for maternal age category (≤19 [referent], 20-29, 30-39, ≥ 40 y), parity (0 [referent], 1, 2, ≥ 3), rural or urban/missing [referent] residence, neighbourhood income quintile (Q1/missing [referent], Q2, Q3, Q4, Q5), pregnancy or birth as a product of spontaneous ovulation & conception, maternal co-morbidities categorized by John Hopkins Aggregated Diagnosis Groups (0-2 [referent], 3-4, 5-31), and 1+ visits to the ED within 365 d before the index pregnancy.

Table S6. Risk of maternal mortality within 42 days after the index delivery hospitalization discharge date (upper), and neonatal mortality within 27 days after the index birth hospitalization discharge date (lower), each by adequacy of prenatal care based on the R-GINDEX. The assessment of SMM was among all livebirths and stillbirths, while that for SNM was limited to livebirths. Counts < 6 are suppressed for privacy reasons.

|  |  |  | **Relative Risk (95% CI)** | |
| --- | --- | --- | --- | --- |
| **Outcome** | **Exposure group** | **No. (%) with outcome** | **Unadjusted** | **Adjusted^a, b^** |
| **Maternal mortality** | No prenatal care  (N = 1923) | 32 (0.01) | 22.11 (6.77 to 72.14) | 12.01 (3.65 to 39.5) |
|  | Inadequate prenatal care  (N = 29,118) | Suppressed | Suppressed | 1.74 (0.58 to 5.19) |
|  | Intermediate prenatal care  (N = 126,968) | 26 (0.01) | 0.78 (0.34 to 1.77) | 0.77 (0.34 to 1.78) |
|  | Adequate prenatal care  (N = 453,482) | 7 (0.01) | 1 (Ref.) | 1 (Ref.) |
|  | Intensive prenatal care  (N = 344,323) | Suppressed | Suppressed | 0.94 (0.54 to 1.61) |
|  |  |  |  |  |
| **Neonatal mortality** | No prenatal care  (N = 1833) | 22 (1.2) | 5.10 (3.34 to 7.81) | 4.47 (2.91 to 6.86) |
|  | Inadequate prenatal care  (N = 28,906) | 42 (0.15) | 0.62 (0.46 to 0.85) | 0.57 (0.42 to 0.78) |
|  | Intermediate prenatal care  (N = 126,327) | 216 (0.17) | 0.73 (0.63 to 0.85) | 0.72 (0.62 to 0.83) |
|  | Adequate prenatal care  (N = 450,538) | 1050 (0.23) | 1 (Ref.) | 1 (Ref.) |
|  | Intensive prenatal care  (N = 343,153) | 416 (0.12) | 0.51 (0.46 to 0.57) | 0.45 (0.40 to 0.50) |

^a^For the outcome of maternal mortality, the model was adjusted for maternal age category (≤ 19 [referent], 20-29, 30-39, ≥ 40 y), parity (0 [referent], 1, 2, ≥ 3), rural or urban/missing [referent] residence, neighbourhood income quintile (Q1/missing [referent], Q2, Q3, Q4, Q5), pregnancy or birth as a product of spontaneous ovulation & conception, maternal co-morbidities categorized by John Hopkins Aggregated Diagnosis Groups (0-2 [referent], 3-4, 5-31), 1+ visits to the ED within 365 d before the index pregnancy, and current stillbirth.

^b^For the outcome of neonatal mortality, the model was adjusted for maternal age category (≤19 [referent], 20-29, 30-39, ≥ 40 y), parity (0 [referent], 1, 2, ≥ 3), rural or urban/missing [referent] residence, neighbourhood income quintile (Q1/missing [referent], Q2, Q3, Q4, Q5), pregnancy or birth as a product of spontaneous ovulation & conception, maternal co-morbidities categorized by John Hopkins Aggregated Diagnosis Groups (0-2 [referent], 3-4, 5-31), and 1+ visits to the ED within 365 d before the index pregnancy.

## Figure S1. Adequate prenatal care and subsequent maternal and neonatal severe morbidity and mortality indicators after birth. Shown is a causal diagram (A) and the timeline for measured variables (B).


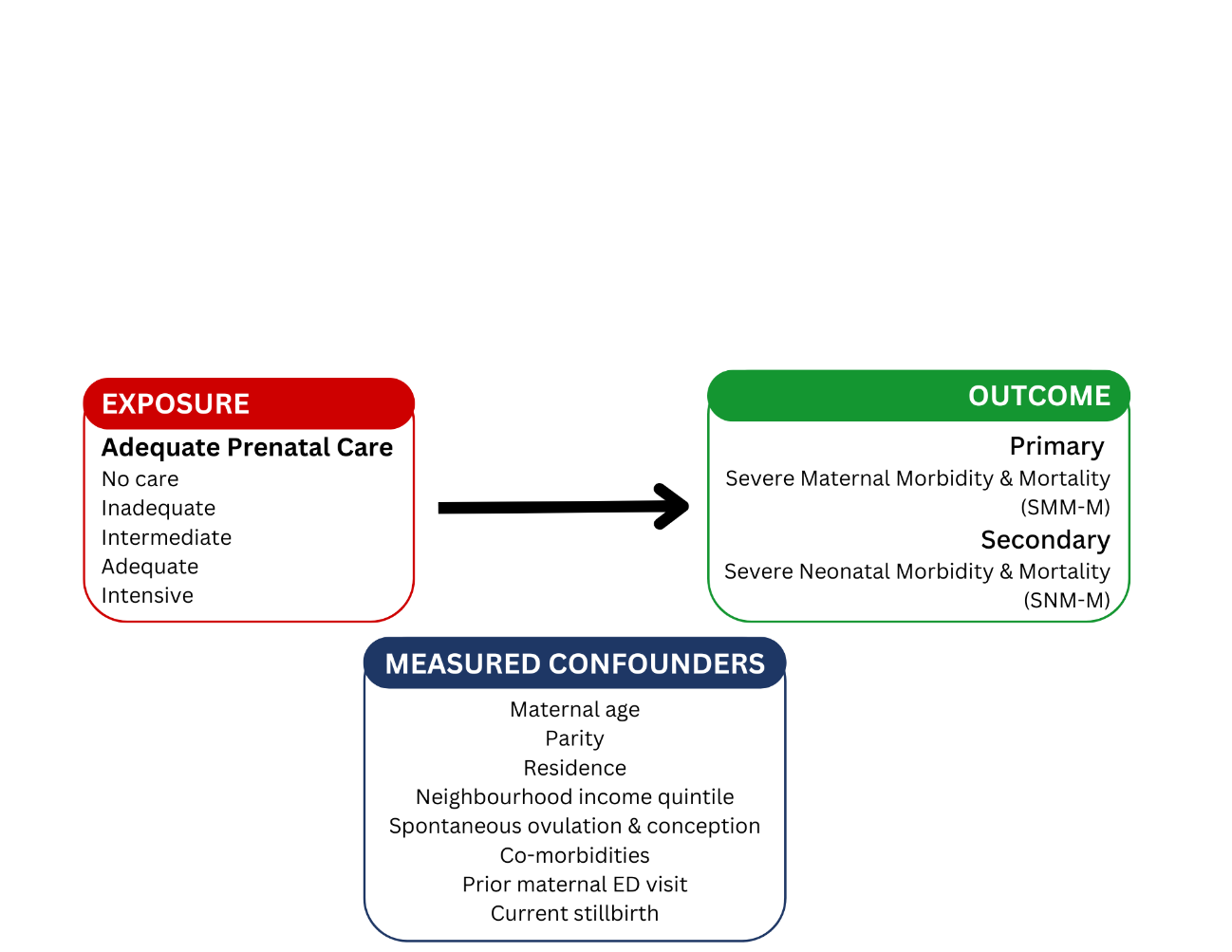


**A**


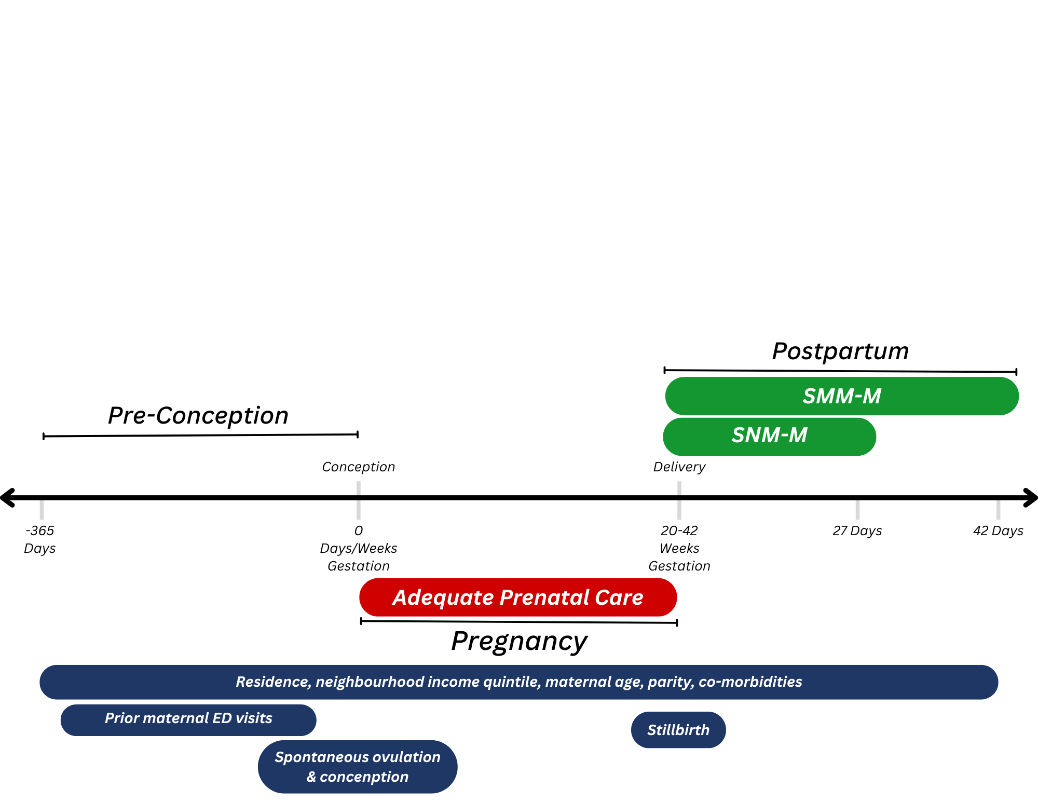


**B**

## Figure S2. Cohort creation.

**Singleton births from April 1, 2012 to March 31, 2020 (N = 1,024,870)**

**Eligible births (N = 955,814)**

**Sufficient Prenatal Care**

N = 924,773

**Insufficient Prenatal Care**

N = 31,041

**Number excluded**:

- Not an Ontario resident: 718
- Invalid IKN or sex in RPDB: 62
- Ineligible for OHIP within 365 d before conception: 65,292
- Warning on female/infant record: 2548
- Gestational age < 20 or ≥ 43 weeks: 436

**Livebirths**

N = 920,018

**Livebirths**

N = 30,739
